# AlacatDesigner—Computational Design of Peptide Concatamers for Protein Quantitation

**DOI:** 10.1021/acs.jproteome.2c00608

**Published:** 2023-01-23

**Authors:** Martin Rusilowicz, David W. Newman, Declan R. Creamer, James Johnson, Kareena Adair, Victoria M. Harman, Chris M. Grant, Robert J. Beynon, Simon J. Hubbard

**Affiliations:** † Division of Evolution, Infection and Genomics, School of Biological Sciences, Faculty of Biology, Medicine and Health, Manchester Academic Health Science Centre, University of Manchester, Manchester M13 9PT, United Kingdom; ‡ Division of Molecular and Cellular Function, School of Biological Sciences, Faculty of Biology, Medicine and Health, Manchester Academic Health Science Centre, University of Manchester, Manchester M13 9PT, United Kingdom; § GeneMill, Institute of Systems Molecular and Integrative Biology, University of Liverpool, Crown Street, Liverpool L69 7ZB, United Kingdom; ∥ Centre for Proteome Research, Institute of Systems and Integrative Biology, University of Liverpool, Crown Street, Liverpool L69 7ZB, United Kingdom

**Keywords:** proteomics, absolute quantitation, protein standards, QconCATs, bioinformatics, peptide surrogates, proteotypic, quantotypic

## Abstract

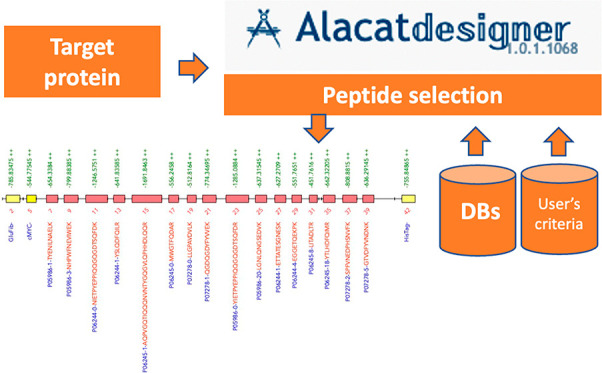

Protein quantitation via mass spectrometry relies on peptide proxies for the parent protein from which abundances are estimated. Owing to the variability in signal from individual peptides, accurate absolute quantitation usually relies on the addition of an external standard. Typically, this involves stable isotope-labeled peptides, delivered singly or as a concatenated recombinant protein. Consequently, the selection of the most appropriate surrogate peptides and the attendant design in recombinant proteins termed QconCATs are challenges for proteome science. QconCATs can now be built in a “a-la-carte” assembly method using synthetic biology: ALACATs. To assist their design, we present “AlacatDesigner”, a tool that supports the peptide selection for recombinant protein standards based on the user’s target protein. The user-customizable tool considers existing databases, occurrence in the literature, potential post-translational modifications, predicted miscleavage, predicted divergence of the peptide and protein quantifications, and ionization potential within the mass spectrometer. We show that peptide selections are enriched for good proteotypic and quantotypic candidates compared to empirical data. The software is freely available to use either via a web interface AlacatDesigner, downloaded as a Desktop application or imported as a Python package for the command line interface or in scripts.

## Introduction

Accurate absolute quantification of proteins remains a challenge in proteomics and requires the use of internal standards of known concentration against which the signal from endogenous proteins can be calibrated. While intact proteins can sometimes be detected directly by mass spectrometry (MS),^1^ the majority of bottom-up proteomics pipelines use an enzymatic digesion step to generate their constituent peptides from which parent proteins can be identified, and ideally, quantified. These surrogate peptides are therefore typically used as a proxy in bottom-up workflows, either as standalone peptides made by direct chemical synthesis (AQUA peptides^2,3^), short representative epitope fragments (PrESTs^4^), as part of a complete recombinant protein (PSAQ^5^), or via a selected subset of representative peptides expressed in a recombinant protein (QCONCATs^6,7^). These peptides can be generated to incorporate stable isotope-labeled amino acids, so they can be distinguished via mass spectrometry by virtue of their mass-to-charge values and hence can be spiked in as an internal standard. In all cases except the most demanding and expensive solution, PSAQs, there is a requirement to select peptides which are best suited to the task. These peptides should be proteotypic,^8,9^ a term developed to refer to the peptides that are routinely detected by MS experiments in a standard LC-MS/MS workflow where the parent protein is present. The condition ensures that if the protein is present at a reasonable concentration in the sample then the peptide will be readily ionized and detected in the gas phase. A number of computational prediction tools have been developed to predict this “detectability” property to support the selection of these surrogates.^10−13^ Moreover, they should be readily cleaved to completion by the proteolytic enzyme used in the experiment, typically trypsin, from both the endogenous protein and the analytical protein standard such as a PSAQ or QconCAT.^7^ If either of these reactions do not go to completion or does not progress with matched kinetics (this is less desirable) then the quality of the subsequent quantitation will be compromised as the peptide bonds flanking the endogenous or standard peptide will not be cleaved to the same extent.^14^ This would lead to errors when estimating quantitative ratios from the extracted ion signals measured in the mass spectrometer. Again, a series of computational prediction tools have been developed to help nominate peptides with amenable cleavage sites,^15^ sometimes in tandem with “detectability”.^16^

Once peptides have been selected and the corresponding standards have been obtained, protein identification and quantitation experiments can be performed, usually via LC-MS/MS. Several suitable MS variations exist, including selective reaction monitoring (SRM), data-dependent acquisition (DDA), and data-independent acquisition (DIA) approaches. DIA-based approaches offer the greatest selectivity and sensitivity, since they are usually targeted toward the peptide precursor ions of interest as well as predetermined fragment ions, all observed at an expected retention time (RT) eluting into the MS. This provides a set of coordinates in terms of RT, precursor ion *m*/*z*, and fragment ion *m*/*z* values, which gives high selectivity. For example, we used this approach to quantify over 1800 yeast proteins using heavy labeled QconCATs and SRM analyses.^17^

Regardless of whether AQUA, QconCAT, or similar technologies are employed, peptide selection remains a constant factor and, equally, the elimination of unwanted missed cleavage artifacts. The latter can be reduced by the use of “spacer” sequences (typically, three amino acids for each of the N and C termini of peptides) between the quantitative peptides, recapitulating the endogenous cleavage context of the protein under study.^18^ These spacers give the greatest chance of harmonizing the peptide release rate, although it must be stressed that the reaction proceeds to completion, irrespective of cleavage context. In the most recent version of the QconCAT technology, multiplexed standards are generated via cell-free synthesis of a recombinant protein, predicated on selection of “Qbricks” that are oligonucleotides encoding paired surrogate peptides for a single protein target. The Qbrick also incorporates short flanking peptides corresponding to the native primary sequence of the endogneous Qpeptides. This supports a synthetic biology approach whereby Qbricks can be readily synthesized, stored, cataloged, and accessed to enable the synthesis of a QconCAT to order; which we term ALACATS (QconCATS “a la carte”).

The selection of the most appropriate peptides for quantitation is therefore important, since sequences can recur in a proteome, be poorly detected, and be confounded by missed cleavages or modifications. Consequently, “proteotypic” peptides that are not shared between proteins and serve as (theoretically) indisputable high-quality markers of their parent protein should be selected.^19^ Criteria for peptide selection can be extended further; not only must peptides uniquely represent the protein of interest but also they must be detectable in the mass spectrometer, preferably with a high signal:noise ratio in order to be distinguishable from low-level background noise. The signal should be well above the limits of detection of the instrument, particularly if the protein in question is in low abundance.

Additionally, although a peptide itself might be deemed proteotypic and is near universally detected if its protein is in the sample, it might not actually be a good surrogate for the protein abundance itself; this concept is referred to as “quantotypy”.^20^ Various effects, ranging from post-translational modifications in vivo, different degrees of miscleavage during workbench digestion, oxidation potential, to gas-phase ionization properties, might all contribute to additional variation in signal detection and quantitation within the mass spectrometer itself. These factors could all vary in different biological or technical circumstances, and all generate deviations in how well individual peptide quantifications correlate with the protein quantifications of actual interest.^21^ This property can be empirically estimated by the variance observed in a peptide’s signal intensity across matched runs with respect to its parent protein.

Identification a priori of those peptides that provide the best correlation with their parent protein is not simple. Resources such as PeptideAtlas,^22^ Massive,^23^ and PRIDE^24^ are a source of proteotypic information, i.e., frequent detection in proteomic experiments. This does not strictly encapsulate “quantotypy” as a concept. One approach to this is simply to select against “antiquantotypic” properties from candidate peptides based on sequence properties. At its simplest, this involves the elimination of peptides containing amino acids known to cause additional variance during mass spectrometry, such as oxidation of methionine or deamidation of asparagine or gluatamine. In such cases, the ion signal attributed to the peptide (and hence protein) is diluted across multiple species (e.g., both the reduced and the oxidized forms of a met-containing peptide), thereby potentially confounding quantification by introducing additional variability. Other tools have attempted to predict ionization properties explicitly, selecting for peptides that ionize well and are readily detected.^11,25^ Our own tool Consequence^10^ does this using a machine learning approach trained on empirical data garnered from multiple proteomics experiments. Similarly, McPred attempts to predict those tryptic cleavage sites at either end of the surrogate peptide which might be incompletely digested.^15^ Many similar tools have been developed which focus on specific aspects of proteotypic design and prediction^11,12,16,25^ or are larger suites which provide a single workspace for design and selection of peptides.^26,27^ Here, we also include a prototype prediction tool that attempts to predict low peptide variance with respect to the protein quantification, via a tool *DoesItFly*, described in the Methods.

AlacatDesigner is therefore an integrated platform that brings many of the proteotypic and quantotypic concepts together in one space. The new fully user-customizable software tool exploits multiple data sources and algorithms in order to support proteomics practitioners in the selection and design of quantitative peptide standards. The tool is showcased with examplars demonstrating utility benchmarked against empirical observation in label-free shotgun experiments and a recent standard for quantification of protein stoichiometries.

## Methods

### Data and Data Sets

To validate the AlacatDesigner tool, we used published yeast proteomics from a series of label-free experiments to measure changes in protein levels at different growth rates in a chemostat cell culture.^28^ Six different dilution rates were used, 0.2, 0.23, 0.27, 0.3, 0.32, and 0.34 h^–1^, in two independent replicate cultures (ProteomXchange/PRIDE: PXD030003). Protein samples were prepared for analysis as described on a Q Exactive HF Hybrid Quadrupole-Orbitrap Mass Spectrometer prior to analysis using MaxQuant. Typically 3200–3500 high-quality protein identifications were obtained across each growth controlling for FDR at 1%. In total, across the 28 raw files analyzed for the 6 growth rates, we identified 40 385 peptides. We discarded peptides with missed cleavages and others which were not present in a theoretical simulated tryptic limit digest, retaining 33 445 peptides available for analysis. We partitioned these peptides into three classes (“Low”, “Medium”, and “High”) with different inherent detectabilities, based on MaxQuant “MS/MS Count” statistics: the number of samples in which the peptide was definitively identified via MS/MS. This was achieved using the 33.3% and 66.6% percentiles of the MS/MS count, with the “High” group corresponding to those peptides with the highest MS/MS counts. These three groups contain 11 265, 10 873, and 11 307 peptides, respectively, and correspond to MS/MS counts of [0–6], [6–25], and [25–822]. The majority of peptides present in the simulated digest were not identified by MaxQuant at all and were placed into a fourth “Not Found” group comprising 143 583 peptides.

### Tryptic Digestion

For tryptic digestion of the yeast PKA protein QconCAT, 50 μg of protein was treated with 0.05% (w/v) RapiGest SF surfactant at 80 °C for 10 min, reduced with 4 mM dithiothreitol (Melford Laboratories Ltd., UK) at 60 °C for 10 min, and subsequently alkylated with 14 mM iodoacetamide in the dark at room temperature for 45 min. Iodoacetamide was quenched with 3 mM dithiothreitol. Protein digestion was performed with 1 μg of Trypsin Gold, Mass Spectrometry grade (Promega, USA), at 37 °C overnight. Tryptic activity was terminated by the addition of trifluoroacetic acid (Greyhound Chromatography and Allied Chemicals, UK) to a final concentration of 0.5% (v/v) and incubated at 37 °C for 45 min. The sample was centrifuged at 13 000 × *g* and 4 °C for 15 min to remove precipitates, and the cleared supernatant fraction was dried to completion by vacuum centrifugation. The sample was resuspended in 97% H_2_0/3% acetonitrile/0.1% trifluoroacetic acid (LC-MS grade) for LC-MS/MS analysis.

### LC-MS/MS

The protein digest was analyzed using an UltiMateTM 3000 RSLCnano system coupled to a Q Exactive HF Hybrid Quadrupole-Orbitrap Mass Spectrometer (ThermoFisher Scientific, UK). The sample was loaded onto the trapping column (ThermoFisher Scientific, PepMap100, C18, 300 μm × 5 mm) using partial loop injection, at a flow rate of 12 μL/min of 0.1% (v/v) trifluoroacetic acid, 2% (v/v) acetonitrile in water for 7 min. The sample was resolved onto the analytical column (ThermoFisher Scientific, Easy-Spray C18 75 μm × 500 mm 2 μm column) using a linear gradient of 3.8% (v/v) acetonitrile/0.1% (v/v) formic acid (Fisher Scientific, UK) to 50% (v/v) acetonitrile/0.1% (v/v) formic acid over 90 min at a flow rate of 0.3 nL/min (2 h program). The data-dependent program used for data acquisition consisted of a 60 000-resolution full-scan MS scan in the orbitrap (AGC set to 3e6 ions with a maximum fill time of 100 ms). The 10 most abundant peaks per full scan were selected for HCD MS/MS (30 000 resolution, AGC set to 1e5 ions with a maximum fill time of 45 ms) with an ion selection window of 2.0 *m*/*z* and normalized collision energy of 30%. Ion selection excluded ions with a +1 charge state and ions with a charge state equal to or greater than +6. To avoid repeated selection of peptides for fragmentation, the program used a 60 s dynamic exclusion window.

### Peptide Class Enrichment

To assess AlacatDesigner’s ability to recommend peptides that were frequently observed experimentally, we assessed the enrichment of the tool’s top recommended peptide in the four peptide classes defined via MaxQuant, either globally or for a selected metric/feature, compared to random selection. For each protein in the yeast proteome with at least 20 tryptic peptides produced by a limit digest, we selected the peptide with the highest (or lowest) metric of interest to establish a “favored” set of peptides for that metric. This resulted in a set of 3520 proteins and 3520 corresponding favored peptides. We then counted the number of peptides present in each group (H, M, L, and N). The raw counts can be misleading; for many proteins there is simply no good choice of peptide or, conversely, any choice of peptide is good. Therefore, to evaluate the performance of the metrics we compared the group counts from the favored peptides to a set of peptides picked at random (in this case, one for each protein) and calculated the log odds ratio between the two methods for each group in turn (i.e., log(*n*-favored/*n*-random)). Thus, a good designer strategy should see a positive enrichment log-odds score for features that select peptides that are proteotypic as judged by MaxQuant identifications in the H, M, and L groups.

### Base Tools and Design Workflow

**Figure 1:**
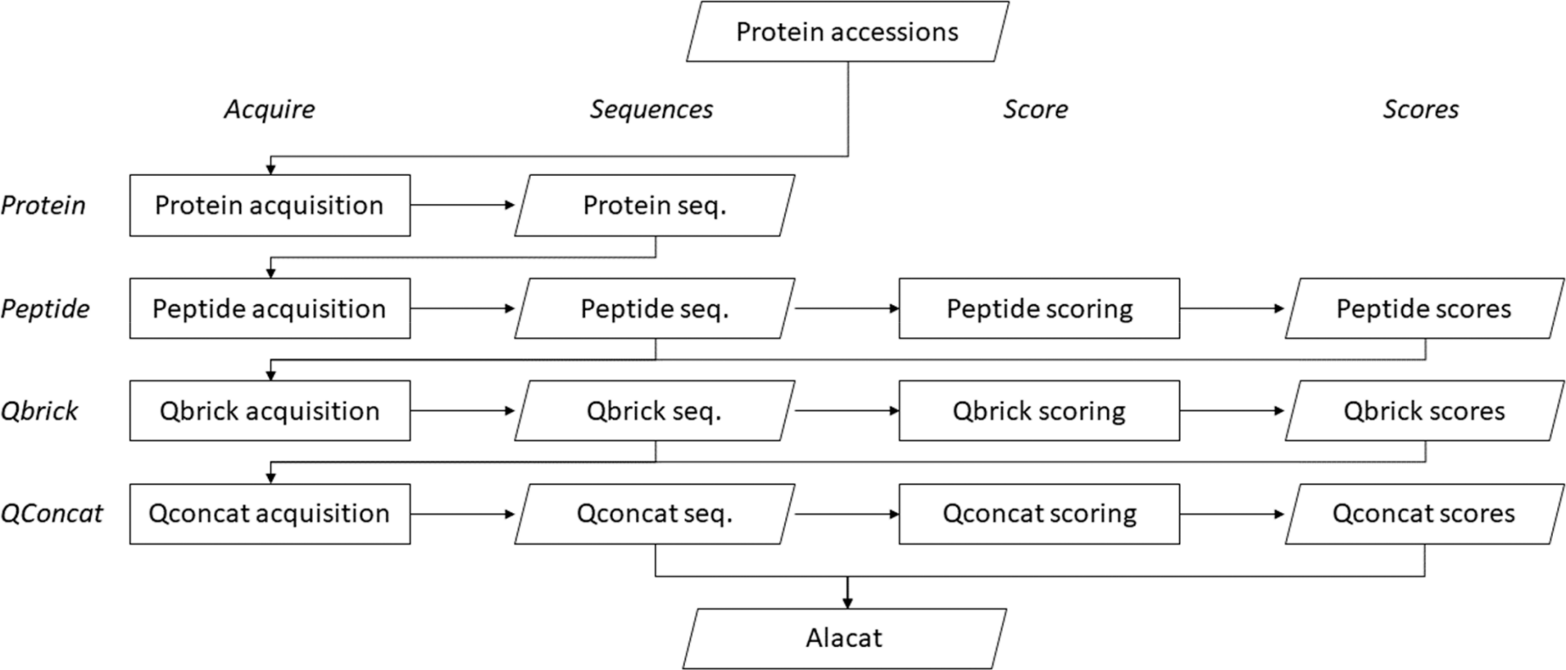
AlacatDesigner workflow. Overall design of the workflow stems from protein accessions provided by the user, via selected peptides and associated Qbricks, that in turn can be combined to form ALACATs (QconCATS).

AlacatDesigner source code is written in extensively commented Python and may be freely explored. It also requires MySQL as an internal backing store to cache results and avoid recomputing metrics for peptides and proteins that have been encountered before. The overall Alacat-Designer workflow is shown in Figure 1.

To initialize the pipeline, the user must present one or more proteins; a variety of formats are accepted including FASTA, CSV, or Uniprot or Ensembl accessions. In the protein acquisition stage, the user input is parsed and translated into the actual protein sequences, downloading data from Uniprot if required.

Following protein sequence acquisition, the sequences are “digested” into their constituent peptides using standard tryptic cleavage rules (KP and RP are not cleaved). Although the user may specify a digester that can provide peptides with alternative proteases, the majority of services available in ALACATdesigner are optimized or produce output that is predicated on tryptic cleavage, and we do not currently recommend use of alternative endopeptidases/peptides. Some of the digestion tools that can be selected (i.e., CONSseQuence, McPred, and PPA) also produce peptide metrics that are retained for progression into the next (scoring) stage.

Finally, further scoring metrics can be assigned to the individual peptides. Subsequently, the two most “quantotypic” peptides are selected per protein, that is, those scoring the highest overall, to progress into the next (Qbricks) stage.

Qbricks are then generated from the peptide sequences. Since the user may choose to generate more than one Qbrick per protein, the Qbricks are represented as sets of Qbricks, or “Qblocks”. Each “Qblock” comprises a set of Qbricks that contains the set of peptides previously selected for a particular protein. The number of Qblocks is therefore equal to the number of proteins initially presented. Similar to the peptides, the Qblocks are scored, and the highest scorers are selected to progress into the final QconCAT stage.

In the final stage, QconCATs are generated via permutation from the Qblocks. Since a user-definable limit on QconCAT length may be present, the QconCATs are represented as sets of QconCATs or “Qmenus”, where each “Qmenu” contains a set of QconCATs that encompass all previously selected Qblocks. Again, these “Qmenus” are scored, and the highest scorers are selected to progress to the user as the final output.

It is important to state that, for simplicity, the workflow has been described in complete (proteins to QconCATs) mode only. Concretely, the user may initiate the workflow at any stage and terminate it at any of the subsequent stages, enabling total user control over the design process where particular criteria or specific peptides are required. For instance, the user may begin with their choice of peptides and end once they have received the peptide assessment scores.

### Scoring Process

During scoring, each subject (peptides, Qblocks, or QconCAT) is scored using user-selectable sets of pertinent metrics to provide a matrix of metrics (columns) against the scored subjects (rows). To accommodate metrics of differing importance and reliability, evaluation follows a simple weighted Boolean model that can be user configured. An assessment (rule) is applied to each metric column in turn. The rule indicates whether a particular value is a PASS or FAIL.

After evaluating the rules, the subjects receive points based on whether or not they passed each metric. This can be represented as a simple linear model
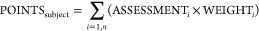
where POINTS is the final score of the subject (e.g., peptide, Qbrick, etc.), ASSESSMENT is the grade of the assessment (1 for PASS and 0 otherwise), and WEIGHT is a user value assigned to the column, representing the importance of this column from a given set from 1 to *n*.

The default weights are orders of magnitude apart, chosen from 6 categories (DISABLED, VERY_HIGH, HIGH, NORMAL, LOW, or VERY_LOW) so that a subject with a single “HIGH” priority “PASS” will always outscore a subject with numerous “LOW” priority passes. The default weights are DISABLED = 0, VERY_LOW = 1, LOW = 10^2^, NORMAL = 10^4^, HIGH = 10^6^, VERY_HIGH = 10^8^). This method allows empirical evidence and expert knowledge to always take precedence over simulations/predictions, which often agree but are not always correct. For example, default values favor expert opinion (e.g., absence of unwanted amino acids such as NG) over a proteotypic prediction tool, though weights are user customizable. A complete list of default weights for each feature is provided in Supporting Information Table S1.

AlacatDesigner also calls external services to generate metrics. Any failures to connect or other errors are recorded, and only valid scores from assessments where both subjects reported a result are considered during comparison. Assessments may also be voided for a particular column, but the user may still choose to view the value. In these cases, the software will report the grade as “INFO” (i.e., not assessed; for information only).

### Tools and Databases

The various scoring metrics (columns) for the peptides are listed in Table 1. All of these are configurable, and the user may incorporate their own metrics by providing the scoring function in code.

**Table 1:** Tools and Services Available in AlacatDesigner^a^

tools and services	
protein “digesters”—input to these is a protein	
Force	user may indicate their own choice of digestion or may enter peptides directly
OpenMS	uses the OpenMS trypsin digestion tool^29^
Trypsin	uses an internal trypsin digestion tool, following standard rules, cleaving after Lys/Arg when not followed by Pro
CONSeQuence^b^	consensus score indicating tryptic peptides with attendant peptide detectability scores (0–4) with 4 being the highest^10^
McPred^b^	tryptic missed cleavage prediction, provides both a N- and a C-terminal score as well as digest peptides^15^
PPA	peptide detectability score predictor^13^ that also returns peptides
scorers—input is a list of peptides (e.g., from a Digester)	
Pride clusters	peptides are mapped to the Pride clusters database,^30^ recording “presence/absence” as a score, with presence indicating direct robust, empirical evidence the peptide is routinely detected
Uniprot proteome^b^	search of selected Uniprot proteomes is conducted, identifying the number of sequence and monoisotopic mass (MMI) repeats; repeat counts of one are favorable, as these indicate no similar peptides exist within the proteome, other than the peptide itself; identities of duplicated peptides are reported (but scored “unfavorable”), which users can elect to ignore; once a proteome has been downloaded, the service is cached for future use
Repeat	submitted sequences are searched for repeats of candidate peptide sequences, returning 1 for no duplicates
Google Scholar search^b^	literature search for the peptide is conducted using Google Scholar, returning the number of hits; favorable values are nonzero; this tool is disabled in the online version as it represents a violation of Google Scholar’s terms of service
Peptide sieve^b^	ICAT ESI, MUDPIT ESI, PAGE ESI, and PAGE MALDI detectability scores are returned;^11^ higher values indicate increased detectability and are favored
Basic analysis	peptide sequence length is reported (presuming very small or large sequences are undesirable), with further information-only outputs also produced: full peptide sequence, terminal type, N and C flanking sequences, start and end positions within protein, protein accession, name and organism
Yolanda^b^	search of the Yolanda-DB database is conducted, a Manchester-based database of detected DIA data predominantly from K562 cells; counts are reported with nonzero counts being favorable, indicating the peptide has empirical evidence it can be identified
NextProt^b^	number of unique hits and number of unique hits including variant in proteins in the NextProt database,^31^ with scores indicating if the peptide exists in other proteins (0 is favorable); this is only available for human proteins
Peptide mass	monoisotopic mass is reported, along with average mass, molecular formula, and amino acid composition; ideally, peptides have midrange values (similarly to length); this is provided for information and is not scored
EliminationChain	number of expert-guided metrics are reported, based around avoidance of unwanted sequence features; these include cysteine (C), methionine (M), and histidine (H) counts; NG, DG, DP, KP, and RP counts; glutamine (Q) start; K or R in linker; complete linker assertions; by default, only peptides breaking the fewest of these rules are selected
Digestible linkers	whether or not the flanking sequences can be digested is determined; result is the number of fragments, with the ideal value being 1 (i.e., no fragmentation)
DeepMSPeptide	peptide detectability scores are returned with values above 0.5 classified as proteotypic peptides^32^
DoesItFly	in-house classifier based on the DeepMSPeptide model (with minor changes) but retrained using the data from local SWATH-MS DIA data sets; detectability scores are returned with values above 0.5 considered to be quantotypic; this classifier can be easily retrained on the user’s own data in the desktop version of AlacatDesigner
PeptideAtlas	peptide features are returned from this database,^22^ returning informative metrics including the relative hydrophobicity, number of genome locations the peptide is found in, number of observations in the database, number of protein samples in which it is detected, and empirical proteotypic score (and its rank in the protein)
IsoelectricPoint	information-only output is produced, which is the estimated isoelectric point of individual peptides

a These are described in two layers: the first accepts protein sequences for digestion; the second takes peptides as a product of the “digesters”.

b This facet depends on an outside service and will fail to function if that service cannot be accessed.

When run for the first time, the online and desktop tools will download the information for a proteome (e.g., for a given species), but subsequently, this information can then be used offline: Uniprot proteome, PRIDE clusters.^30^

The following facets depend on external programs and are not currently shipped with Alacat-Designer: DeepMSPeptide and PeptideSieve, though they are available in the online version. For interested parties, we provid documentation for integration of services should users require them independently, described on the Bitbucket site. A further two external services, Trypsin and IsoelectricPoint, are provided with the standard download. These are two simple scripts for calculating a list of tryptic peptides from a FASTA file and for estimating the isoelectric focusing point (pI) from an amino acid sequence.

We include here a novel quantotypic peptide predictor, *DoesItFly*, which is based on the DeepMSPeptide model^32^ but retrained using DIA SWATH data obtained from over 331 independent acquisitions of K562 cell line lysate. The classifier was trained on 4638 peptides analyzed with seaMass^33^ partitioning peptides into two classes, low variance and high variance, and is currently still in development, although its overall accuracy exceeds 80%.

There is only one default metric for Qbricks, which favors Qbricks presenting a “natural” peptide order for adjacent peptides (i.e., the peptide order resembling the original protein). There are no default metrics for QconCATs; however, the user may introduce their own if they wish.

## Results and Discussion

**Figure 2:**
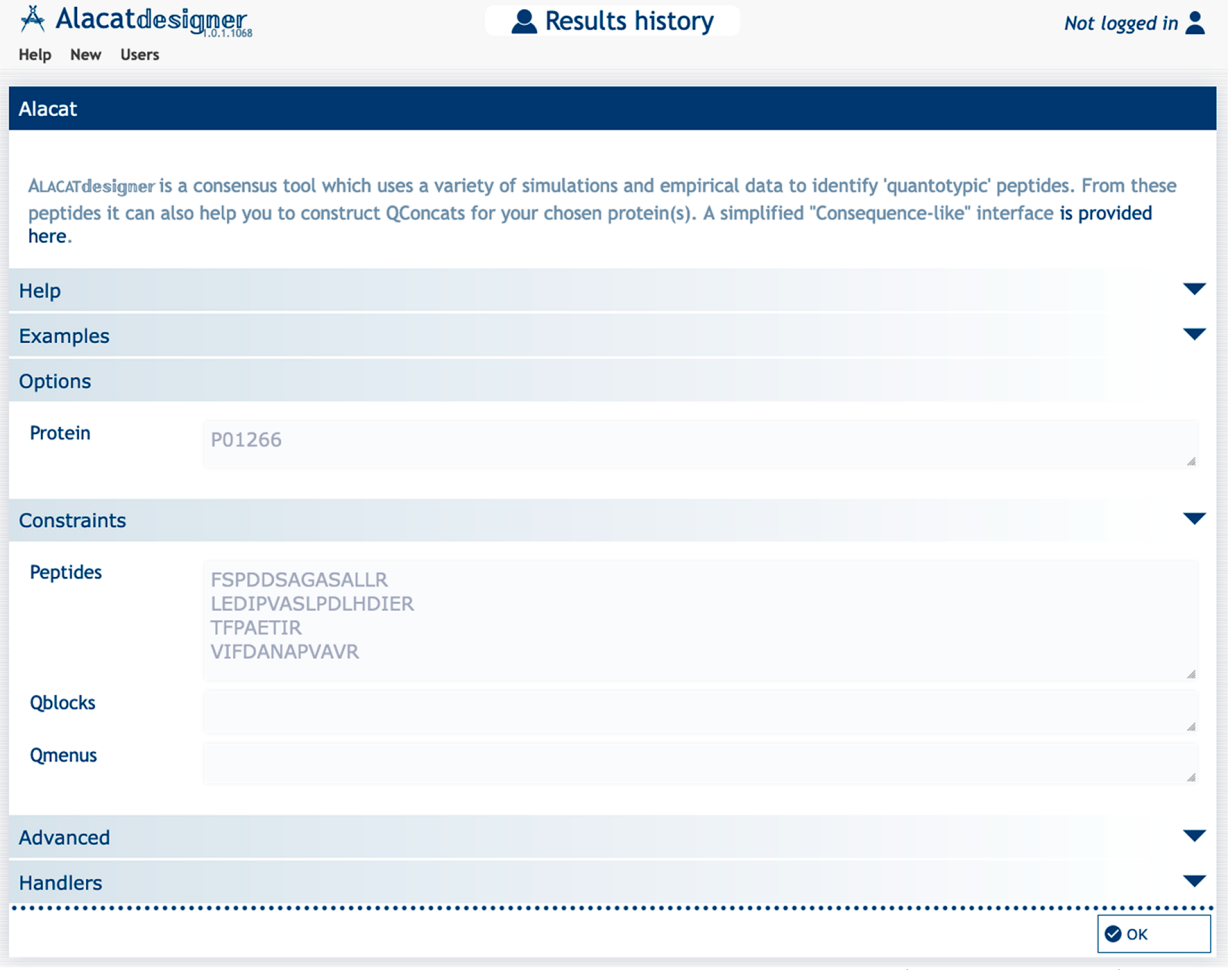
AlacatDesigner web tool interface. Users can enter protein names (UniProt identifiers) or FASTA sequence files in the options box, constrain these to certain peptides, and customize selection criteria via the Handlers option. Examples are provided which illustrate the use of the tool for a variety of purposes.

AlacatDesigner is a graphical user interface-driven suite of services for the selection of surrogate peptides for use in targeted proteomics experiments. The service is available directly through a web browser, where users begin by inputting a protein or set of proteins, as shown in Figure 2. Users can either input UniProt identifiers or supply FASTA-formatted sequences. Appropriate constraints can then be selected to limit the analysis to a subset of peptides or ALACAT-specific criteria. The Handler option allows each selected service to be customized, including their priority (DISABLED, VERY_HIGH, HIGH, NORMAL, LOW, or VERY_LOW) as well as service-specific criteria. Each service is described in some detail in the Handler section. Finally, the “OK” button starts the selection process. Output is returned in the form of a large table of peptide lists for each protein, along with outputs from the services. Peptides are ranked in order based on the user-selected criteria. A comprehensive Help facility is provided along with several examples, and the interface is therefore intuitive.

To demonstrate the efficacy of the peptide selection tool, we present two use cases representing a general and specific example from yeast proteomics. The first examines AlacatDesigner’s ability to pick peptides in general which are “proteotypic” and are frequently observed in a typical proteomics experiment using yeast as a test system. Since AlacatDesigner uses data and tools that have experimental yeast proteomic data (e.g., CONSeQuence, McPred, PRIDE clusters, etc.), we expect it to perform well, but for clarity, we emphasize that none of these tools have been explicitly trained on the specific data set considered here. Furthermore, “proteotypy” is not the sole consideration for selection of surrogate peptides in a targeted MS experiment, and hence, AlacatDesigner also supports selection of peptides that are also likely to be “quantotypic” through simple sequence-based expert rules and also from the *DoesItFly* predictor. Some of these features are illustrated in the next section.

**Figure 3:**
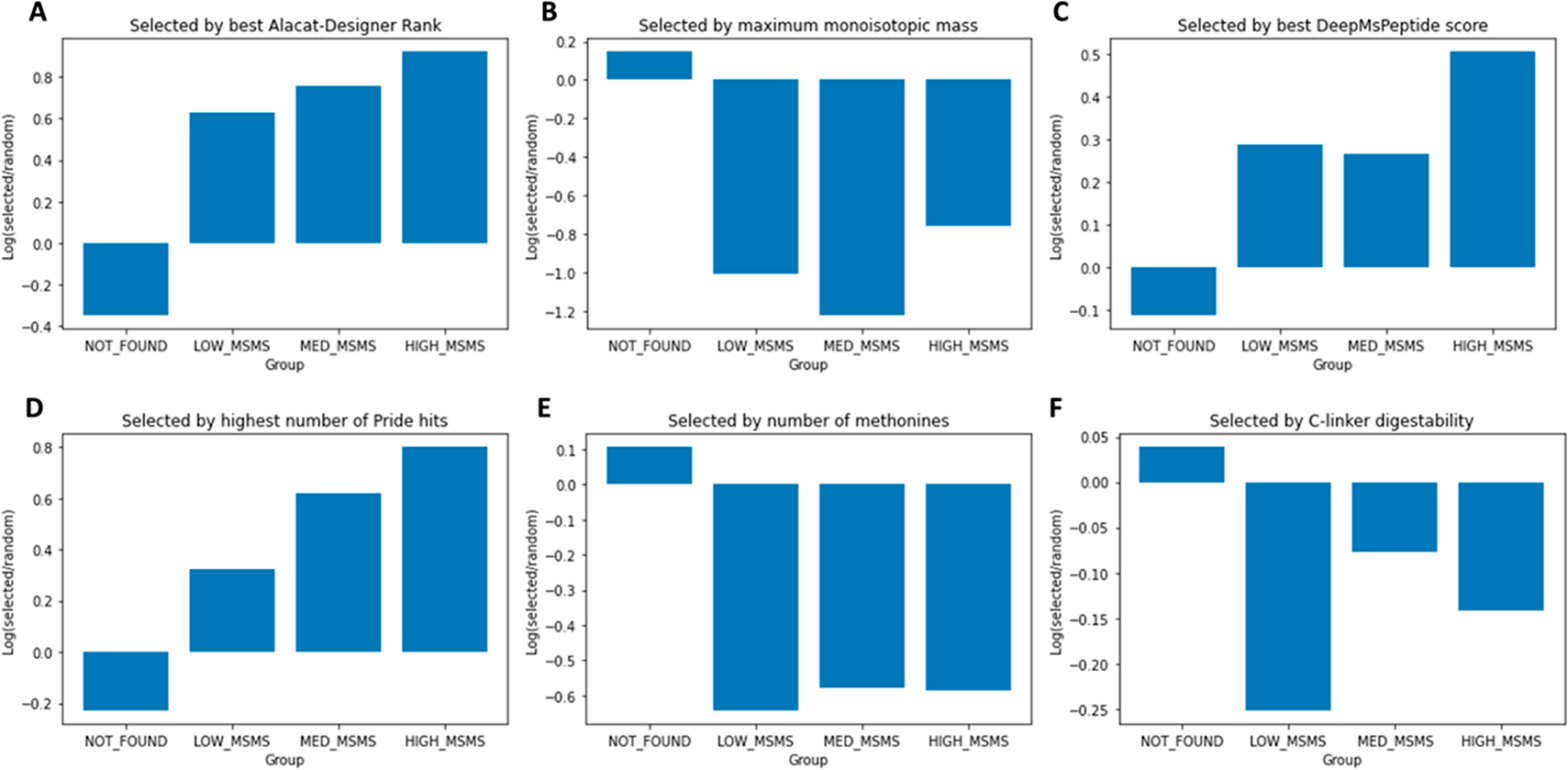
Log-odds enrichment scores for AlacatDesigner-selected peptides for different metrics in proteotypic peptide classes. Selection criteria which favor proteotypic peptide selection will result in positive log-odds scores compared to random peptide selection. (A) Overall AlacatDesigner rank. (B) Maximum peptide mass. (C) DeepMSPeptide prediction score. (D) Number of hits from a PRIDE cluster. (E) Number of methiones in the peptide. (F) Presence of an R or K in 3 amino acids C-terminal to the peptide.

We took a set of label-free shotgun proteomics runs acquired on a QExactive HF orbitrap instrument for biological replicate yeast cultures grown at six different growth rates.^28^ Peptides identified by the MaxQuant search engine were then partitioned into proteotypic categories based on their observance across 28 raw files covering the 6 growth rates (see Methods): High, Medium, Low, and NotFound. We then calculated the log-odds ratio of observing peptides in each category using the AlacatDesigner top selections for each protein compared to random. These log-odds scores are plotted in Figure 3 for six example metrics.

The first example criteria, the overall AlacatDesigner rank (Figure 3A), shows a positive enrichment for our three empirical proteotypic classes (High, Medium, and Low) but not for the NotFound category, as would be expected. Additionally, the log-odds score increases from Low to High proteotypic peptide categories, consistent with their definitions: High being the most frequently observed peptides that were directly detected in over 66% of runs, as opposed to inferred from MaxQuant’s matching-between-runs feature. This is reassuring and demonstrates that the tool’s top selection is enriched for peptides that are frequently detected. Similarly, undetected peptides are infrequently selected.

As a control, Figure 3B shows a deliberately poor metric, the maximum peptide mass in the protein, to illustrate the absence of bias. This is indeed the case, with no enrichment in any of the proteotypic peptide categories and a small positive enrichment in nondetected peptides. This makes good sense since high-mass peptides would be less likely to be detected.

The third and fourth metrics are positive ones: an external proteotypic predictor, DeepMSPeptide, and presence in a PRIDE cluster of empirical common peptides observed in public domain yeast data sets. Again, our proteotypic peptide classes are enriched, and the NotFound category is depleted, with the greatest enrichment in the “High” category.

Figure 3E illustrates a challenge when selecting surrogate peptides containing methionines, oxidation of which can split the MS signal into two forms, weakening detectability, and if standard and analyte oxidize differently can lead to inaccurate quantification. Interestingly, selecting the presence of methionines alone appears to be a poor choice, increasing the chances above random of failing to see the peptide and decreasing the changes of it being a good proteotypic choice. This feature is also anticorrelated with our DoesItFly quantotypic predictor (see Supporting Information, Table S2). Despite this, we note that many laboratories often select methionine-containing peptides for QconCATs, potentially because choices are restricted.

Finally, we examined a metric linked to digestion properties (Figure 3F). Although many practitioners use spacer peptides replicating the native sequence, this could introduce additional basic residues in to the spacer and create disfavored dibasics or additional unwanted tryptic cutsites.^14^ This would disfavor efficient tryptic cleavage of the selected peptide of interest, and indeed, we observe that the presence of a K or R in the C-terminal native sequence in the next three amino acids is a poor selection criterion, as these peptides are depleted for proteotypic classes.

Although AlacatDesigner is not intended to replace any existing prediction tools, we assessed the individual features for their performance using the log-odds enrichment approach using a standard “leave-one-out” approach. In all cases, we observed a modest reduction in the log-odds score for each assessed feature based on a 10-fold simulation picking random peptides (see Supporting Information, Table S3). Unsurprisingly, the feature that had the largest loss on log-odds enrichment was the Rank: Proteotypic Score from PeptideAtlas,^22^ an empirical measure of observability from this public database. We caution against overinterpretation here since the weighting scheme was left unchanged. To overcome this, we also assessed the power of individual features using the log-odds metric, and again, empirical observation features were powerful, with the existence in PRIDE clusters being the most potent (see Supporting Information, Table S4). Collectively, these observations point to empirical observation in a public domain database as the clearest single predictor for surrogate peptide selection, though this does not directly value quantotypic features. We note that some of the selectable features are partly correlated (Supporting Information, Table S2), but predominantly they are not.

Collectively, these example cases demonstrate that the tool selects peptides by default that are proteotypic and ideally quantotypic, though this process remains imperfect. Indeed, user selection and knowledge can often take precedence, and this is supported in the tool. We caution too that much of the empirical data collected is dominated by orbitrap instruments, which may well suit many users but not those operating on other instruments, who may prefer to switch off predictors they consider to be biased.

To illustrate the tool in action, we have used AlacatDesinger to assist quantification of a single protein complex in yeast of local biochemical interest, intended to support the determination of protein subunit stoichiometry via absolute quantitation. Peptide standards are increasingly being used as a tool to quantify stoichiometries within protein complexes,^34,35^ where alternate proteoforms form part of an additional challenge for peptide selection and absolute quantification. As a case study, we tested the utility of the software to design standards for the widely studied cAMP-dependent protein kinase (PKA) from *Saccharomyces cerevisiae*. This kinase is a tetrameric complex, comprising two regulatory subunits (encoded by *BCY1*) and two catalytic subunits (encoded by either *TPK1*, *TPK2*, or *TPK3*).^36^ To generate an ALACAT representative of these four subunits, UniProt accession numbers P05986, P06244, P06245, and P07278 were presented to the AlacatDesigner. As the user can specify peptides of their choosing, we also included two peptides per subunit that had been detected in a recent chemostat quantification study^28^ in order to test the agreement between the software- and the user-selected peptides. In this case, user selection was based on frequency of observation and the quality of spectral identification. The AlacatDesigner output provides a number of evidence columns with scores and rankings based on many sources of proteomics data. Other information such as N- and C-terminal flanking sequences and repetitions in the proteome are also included, which are useful when considering alterations that may need to be made based on digestible linkers or whether a peptide is specific to one protein. A refined output is shown in Table 2, highlighting each peptide selected by the user (U) or the software (S) and a selection of rankings as listed in Table 1. User-selected peptides were consistently ranked by the software within the top five peptides per protein accession, indicating the ability of the software to select peptides that are readily detectable by mass spectrometry. This gives high confidence that the software can generate peptides that would also be chosen by an “expert” in their field to quantify their protein(s) of interest.

**Table 2:**
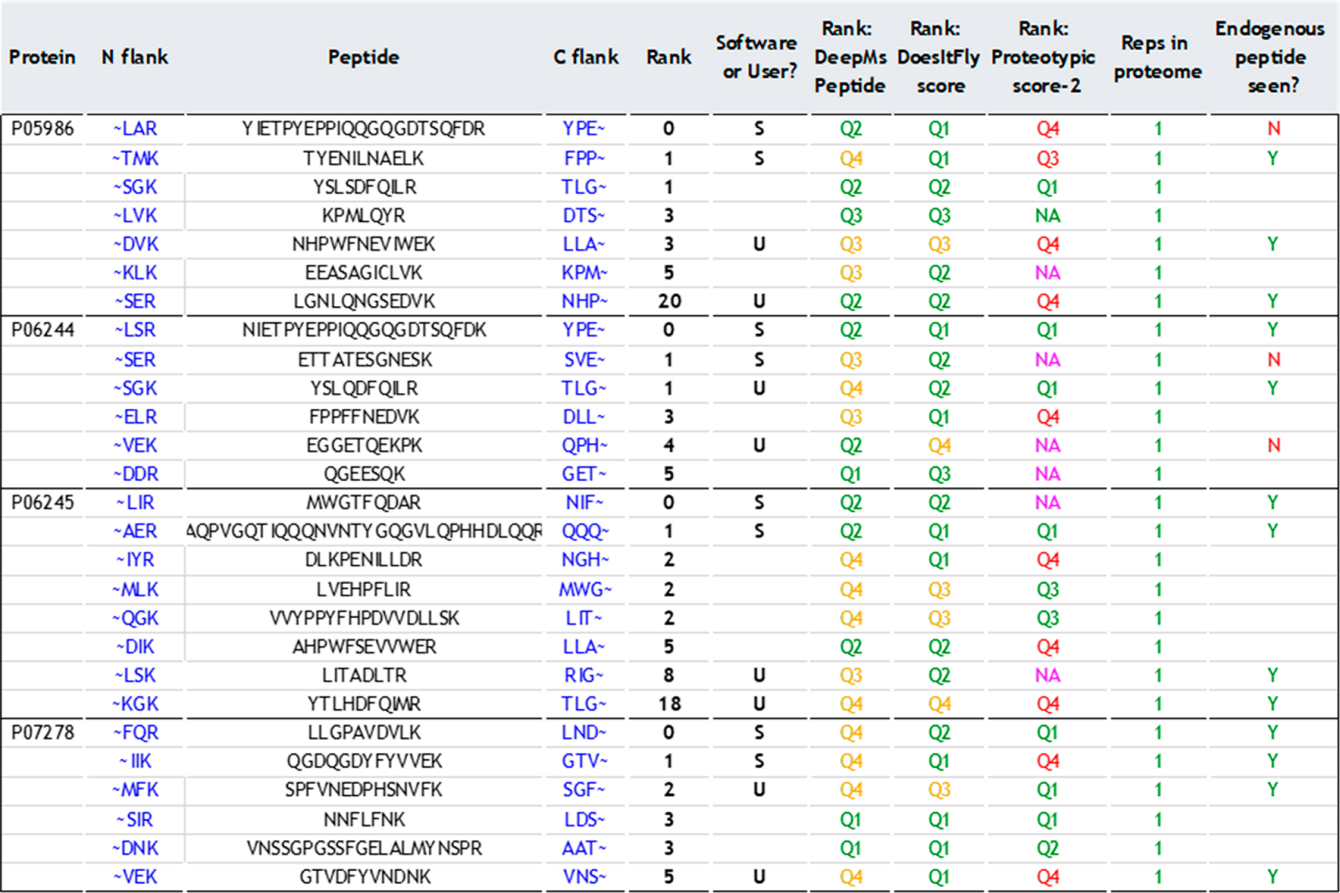
Refined Output Table from AlacatDesigner^a^

a For each of the four proteins selected, the full sequence and flanking regions of the top-ranked candidate peptides along with the four peptides used in the ALACAT (indicated by an S or U in the 6th column; U indicates where User knowledge/expertise was applied; S indicates AlacatDesigner ranking). Three representative features are shown and the attendant quartile ranking according to AlacatDesigner, where Q1 is the top-rated quartile. In the final column, for those peptides selected, Y indicates that the endogenous yeast peptide was also detected and a quantitative value could be derived.

**Figure 4:**
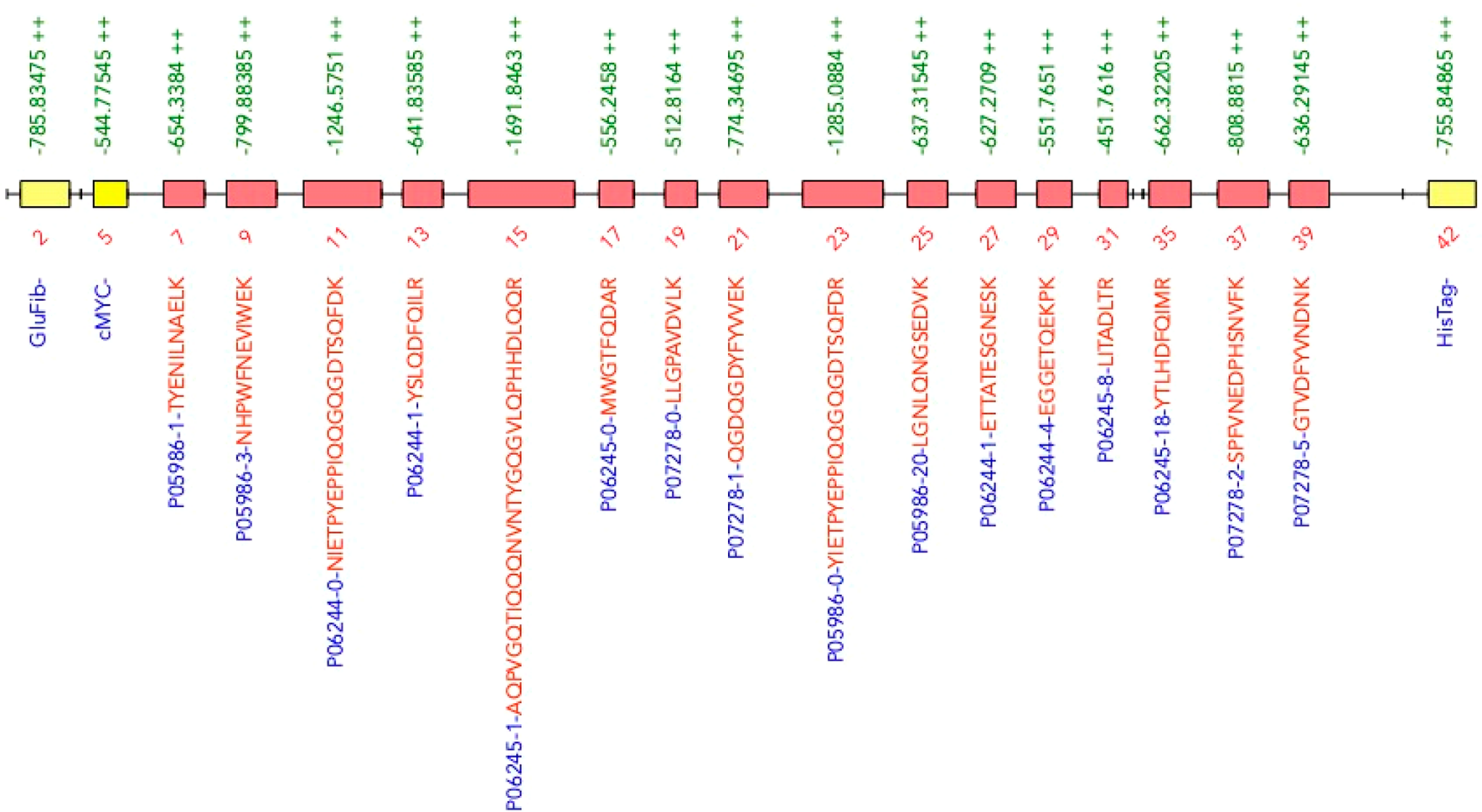
ALACAT peptides visible via mass spectrometry. Peptides T7–T40 represent the designed peptides (with flanking sequences omitted) , while T2, T5, and T42 are the normalization or purification peptides. These are filled in red or yellow, respectively. All bar T27 were detected by LC-MS/MS.

As a proof of principle, the ALACAT generated by AlacatDesigner was assembled as a short ALACAT and expressed and labeled by wheat germ cell-free synthesis (CFS).^37^ The purified construct was then digested with trypsin and analyzed via LC-MS/MS, as described in the Methods. Fifteen out of 16 peptides present in the ALACAT were detected as well as the glu fibrinopeptide (GluFib), c-Myc, and HisTag peptides included for normalization or purification purposes (Figure 4). This shows how AlacatDesigner offers a platform to integrate predictions and user preference for selecting peptides that are detectable by an MS instrument with only one failure. As the underlying data in external repositories can change, we note this peptide is now ranked seventh by AlacatDesigner, so it might have not been selected now. Indeed, the current second-choice peptide (YSLQDFQILR) is observed in our yeast growth rate data set^28^ and might have been a better choice in this one instance.

We also examined AlacatDesigner’s performance on a published study that critically considered the QconCAT as an analytical approach for protein quantification,^38^ illustrated on a use case for Human Thyroglobulin. This study highlights many of the challenges involved in peptide selection for accurate quantitation and reported issues with recombinant standards such as QconCATs in relation to peptide-only and whole-protein quantifications, though recommend recombinant approaches above labeled peptide-only standards. Since this example case has examined peptide performance in some detail, we used AlacatDesigner to evaluate their selection of surrogate peptides. This example case is also available on the AlacatDesigner Web site.

Human Thyroglobulin was presented to AlacatDesigner’s protein input by means of its Uniprot accession, P01266. The user-chosen peptides were selected to match those specified in the original paper and presented to AlacatDesigner’s peptide input: FSPDDSAGASALLR, LEDIPVASLPDLHDIER, TFPAETIR, VIFDANAPVAVR, VILEDK, SQAIQVGTSWK, GGADVASIHLLTAR, and EFSELLPNR.

**Figure 5:**
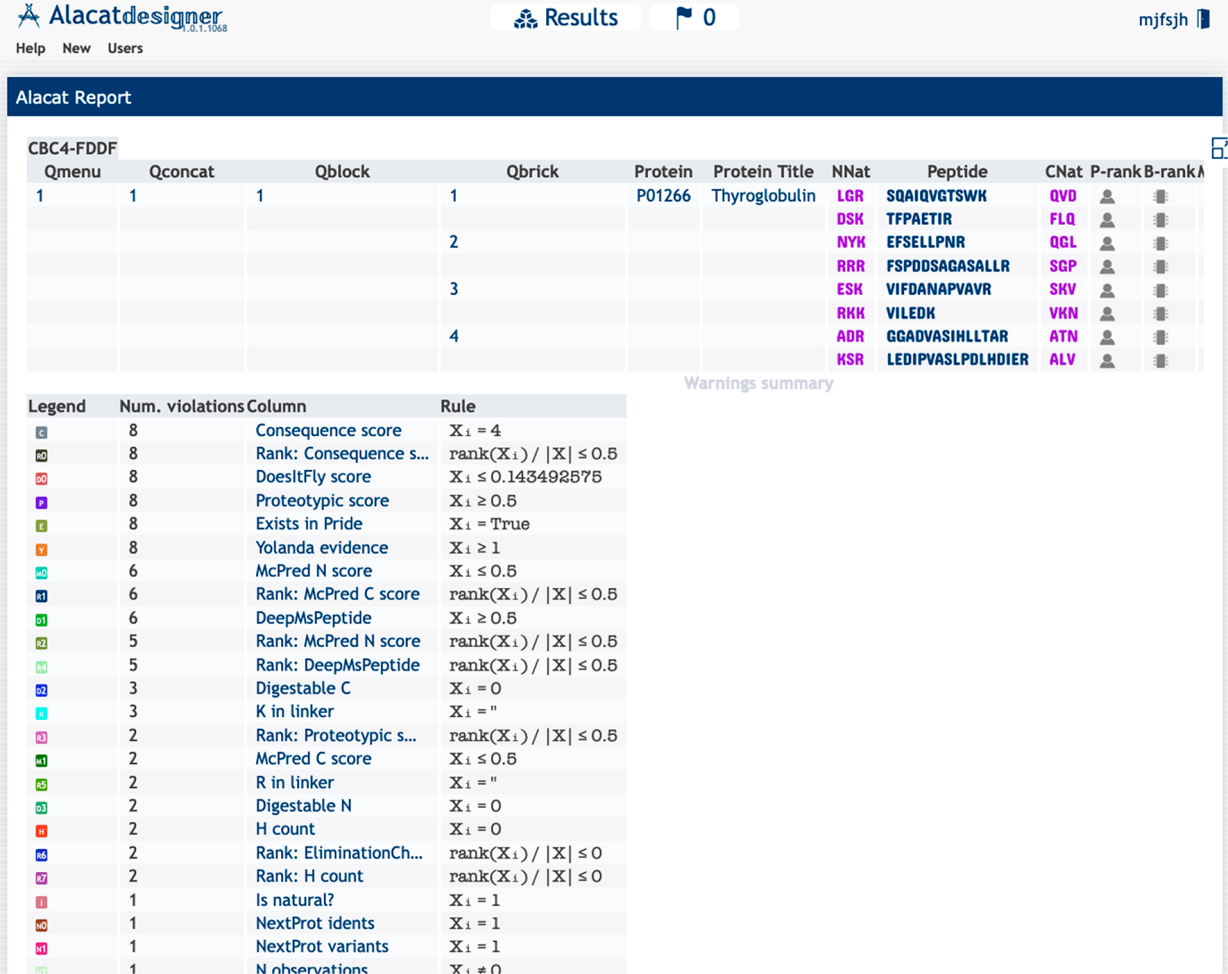
Example AlacatDesigner results. Usecase illustrates a screenshot from the example for human thyroglobulin, Uniprot id P01266, with results pointing to warnings for most peptides in the lower half of the screen. All eight peptides fail on the CONSeQuence filter and six on the McPred missed cleavage score.

All other parameters were left as defaults. For this set of peptides, all of them have some warnings identified in the AlacatDesigner output, Figure 5. Although this is common, none of them were recommended by CONSeQuence or any other proteotypic predictor, and none were included in the 14 that passed AlacatDesigner’s filter. Notably, some of them have very poor sequence contexts at their N- and C-terminal cleavage sites, which would promote missed cleavage and lead to signal loss and variance. For example, VILEDK, as illustrated in Figure 5, has additional tryptic candidate sites in the linker regions, suggesting it would be a very poor quantotypic peptide (it is ranked 53rd by AlacatDesigner overall). Indeed, the authors of the study report numerous issues with cleavage efficiency in support of this, highlighting the utility of AlacatDesigner to flag issues of this nature.

## Conclusions

We present here AlacatDesigner, a customizable and user-friendly tool for the selection of candidate peptides to be used as surrogates in quantitative studies of their parent proteins in mass spectrometry experiments. The tool is designed as a platform to integrate both user and automated selection for proteotypic and quantotypic features and offers a number of reports and views over the peptide-level data. AlacatDesigner is available to use online at https://monod.ls.manchester.ac.uk/alacat/. Additionally, we provide source code so users can add their own tools and services to the platform via a stand-alone desktop version that can be downloaded from the Python Package Index (PyPi): python -m pip install alacat.

Please note that AlacatDesigner requires Python 3.8 and MySQL to run. Code is available from bitbucket via https://bitbucket.org/mjr129/alacat

## Supporting Information Available

Full default weighting scheme used in AlacatDesigner; feature correlation metric; leave-one-out performance assessment; individual feature log-odds enrichment (XLSX)
